# O004: Improved hand hygiene compliance after eliminating mandatory glove use for patients on contact precautions

**DOI:** 10.1186/2047-2994-2-S1-O4

**Published:** 2013-06-20

**Authors:** A Cusini, D Nydegger, T Löhri, K Mühlemann, J Marschall

**Affiliations:** 1 Clinic of Infectious Diseases and Hospital Epidemiology, University Hospital Bern, Bern, Switzerland; 2 Division of Infectious Diseases, Washington University School of Medicine, St. Louis, USA

## Introduction

The requirement to wear gloves when caring for patients on contact precautions may cause personnel to neglect hand hygiene before aseptic procedures, thereby increasing infection transmission.

## Objectives

The aim of this study was to assess the compliance with hand hygiene before and after eliminating mandatory glove use for patients on contact precaution at our institution.

## Methods

We assessed hand hygiene compliance of HCW taking care of 50 adult patients colonized with MDR microorganisms before (2009) and six months after (2012) eliminating the mandatory wearing of gloves. This policy change was implemented by our infection control team and communicated to all hospital floors. Along with this policy change, we routinely provided hand hygiene training to HCW on all floors. Hand hygiene observation was performed by two trained infection control nurses during routine care using a standardized questionnaire.

## Results

We observed 426 hand hygiene indications before the policy change and 492 six months after policy change. Compared to 2009, we observed a significantly higher compliance with hand hygiene in patients colonized with MDR microorganism in 2012 [85.4%, (95% CI 82.2-88.5) versus 51.9 %, (95% CI 47.1-56.6); p< 0.001]. In particular, compliance improved before performing invasive procedures: [72.0% (95%CI 61.6-82.4) vs. 23.9% (95%CI 14.8-32.9); p< 0.001] , and before patient contact [76.7% (95%CI 68.9-84.5) vs. 32.3 (95%CI 24.0-40.5); p<0.001]. Hand hygiene compliance *after* patient contact remained high [93.5% (95%CI 89.2-97.9) in 2012 vs. 94.3% (95%CI 89.8.2-98.8) in 2009; NS]. During the same period, we observed a smaller increase in hand hygiene compliance for the entire hospital (17.5% hospital-wide versus 33.5% in patients on contact precautions).

## Conclusion

Eliminating mandatory glove use in contact with isolated patients increased hand hygiene compliance, particularly before invasive procedures and before patient contacts. Glove use may cause healthcare workers to bypass hand hygiene. The potential impact on the risk of MDR organism transmission should be determined next.

## Disclosure of interest

None declared.

